# Super-resolution fluorescence microscopy studies of human immunodeficiency virus

**DOI:** 10.1186/s12977-018-0424-3

**Published:** 2018-06-08

**Authors:** Jakub Chojnacki, Christian Eggeling

**Affiliations:** 1 MRC Human Immunology Unit, Weatherall Institute of Molecular Medicine, University of Oxford, Oxford, OX3 9DS UK; 2 Institute of Applied Optics, Friedrich-Schiller-University Jena, Max-Wien Platz 4, 07743 Jena, Germany; 3 Leibniz Institute of Photonic Technology e.V., Albert-Einstein-Straße 9, 07745 Jena, Germany

**Keywords:** Human immunodeficiency virus, HIV-1, Super-resolution microscopy, Nanoscopy, Fluorescence

## Abstract

Super-resolution fluorescence microscopy combines the ability to observe biological processes beyond the diffraction limit of conventional light microscopy with all advantages of the fluorescence readout such as labelling specificity and non-invasive live-cell imaging. Due to their subdiffraction size (< 200 nm) viruses are ideal candidates for super-resolution microscopy studies, and Human Immunodeficiency Virus type 1 (HIV-1) is to date the most studied virus by this technique. This review outlines principles of different super-resolution techniques as well as their advantages and disadvantages for virological studies, especially in the context of live-cell imaging applications. We highlight the findings of super-resolution based HIV-1 studies performed so far, their contributions to the understanding of HIV-1 replication cycle and how the current advances in super-resolution microscopy may open new avenues for future virology research.

## Background

The direct observation studies of biological systems via fluorescence microscopy (FM) is an invaluable tool of scientific discovery thanks to its ability for dynamic analysis of multiple specifically labelled molecules of interest. In the field of virology, fluorescence microscopy has enabled researchers to track the virus particle movements through the cells and probe for the co-localisation with cellular components greatly contributing to our understating of the virus replication cycles. However due to the fundamental physical barrier associated with the diffraction limit of visible light the resolution of conventional fluorescence microscope is theoretically limited to ~ 200 nm in the focal plane (*xy*) and ~ 600 nm along the optical axis (*z*) [1] and in fact it is often even lower in non-ideal conditions of actual experiments [2]. Hence the analysis of objects smaller than this limit by conventional FM cannot yield any information about their details. Since viruses are mostly smaller than 200 nm, this makes the studies of virus architecture and the distribution and dynamics of molecules within the individual sites of virus-cell interactions impossible using this method. Therefore for many decades visualisation of subviral details was performed solely via electron microscopy (EM) based methods which became a de facto gold standard for virus imaging. EM and in particular the advanced implementation of EM such as cryo electron tomography (cryo-ET) has yielded invaluable insights into the minute details of virus structures. These are discussed in the accompanying review by Mak and de Marco [3]. However, as is the case with all scientific tools, EM studies carry specific drawbacks. In particular, EM approaches require laborious preparation of biological samples (fixation or freezing) thus making it unsuitable for study of dynamic processes during virus-cell interactions.

This technological impasse for virology studies has changed dramatically with the development of super-resolution fluorescence microscopy (SRFM) or nanoscopy techniques that work around the diffraction limit of light to improve the resolution (for in-depth reviews please refer to [4–6]). While these techniques can now routinely offer a spatial resolution of 10–100 nm the field is constantly evolving with most recent advances indicating that a resolution of down to 1 nm is now achievable [7]. These capabilities represent a powerful approach that combines increased resolution that can resolve virus substructures with all advantages of FM. These include labelling specificity, non-invasive live-cell imaging and higher throughput making SRFM an ideal tool for in-depth studies of subviral architecture and virus-cell interactions.

**Fig. 1:**
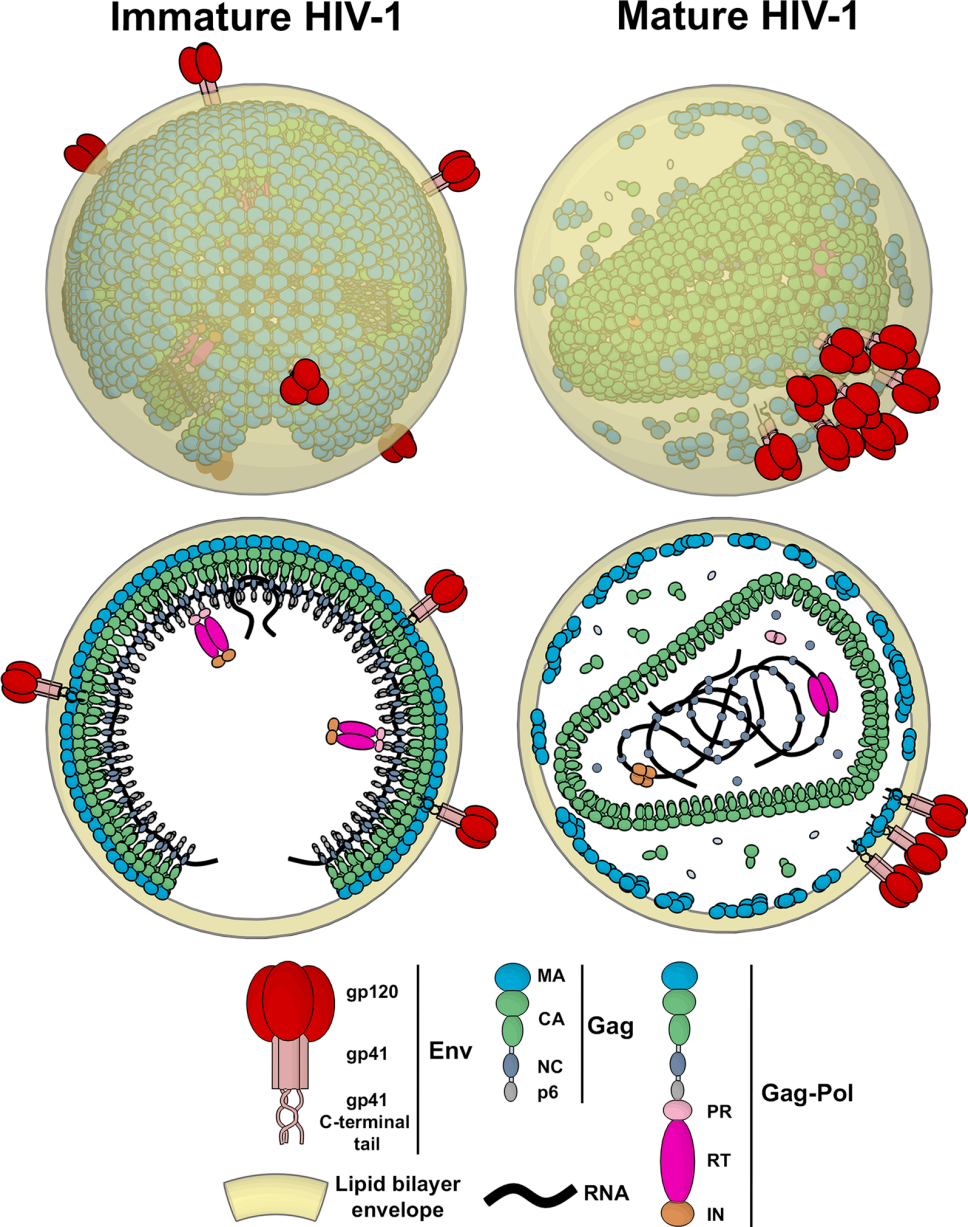
Schematic structure of mature and immature HIV-1 particles with lipid bilayer envelope, Env, Gag and Gag-Pol (with their respective domains) and RNA as labelled. HIV-1 is an enveloped retrovirus with a diameter of 120–140 nm. It is comprised of ~ 2400 Gag polyprotein molecules, which assemble into non-infectious immature virus. Viral enzymes are packaged into the virus as part of the Gag-Pol polyproteins at ~ 1:20 ratio. During assembly and budding 7–10 copies of trimeric fusion glycoprotein Env are incorporated into the lipid viral envelope, along with many host and viral accessory proteins such as Vpr, Vif and Vpu (not shown). Following maturation, the individual domains of Gag (matrix (MA), capsid (CA), nucelocapsid (NC) and p6), Pol [protease (PR), reverse transcriptase (RT) and integrase (IN)] are released and together with Env and RNA undergo reorganisation forming a mature fully infectious virus particle

SRFM studies have provided a number of ground breaking insights into retroviral replication cycle. However, to date these studies have almost exclusively focussed on Human Immunodeficiency Virus Type 1 (HIV-1) (Fig. 1). This is due the fact that over 30 years of intense research into this important human pathogen has already provided a detailed understanding of virus replication cycle. This, in turn, provided guidance and well characterised reagents towards the design of SRFM studies aiming to fill the gaps in the knowledge of HIV-1 biology. In this review we outline the principles of SRFM techniques, and guide the reader through their advantages and disadvantages for virological studies especially in the context of the live cell imaging. Finally, we highlight the findings of SRFM-based HIV-1 studies performed to date, how they have contributed to our understanding of HIV-1 replication cycle and spread and discuss possible future directions in this field.

## SFRM techniques in virus research

**Fig. 2:**
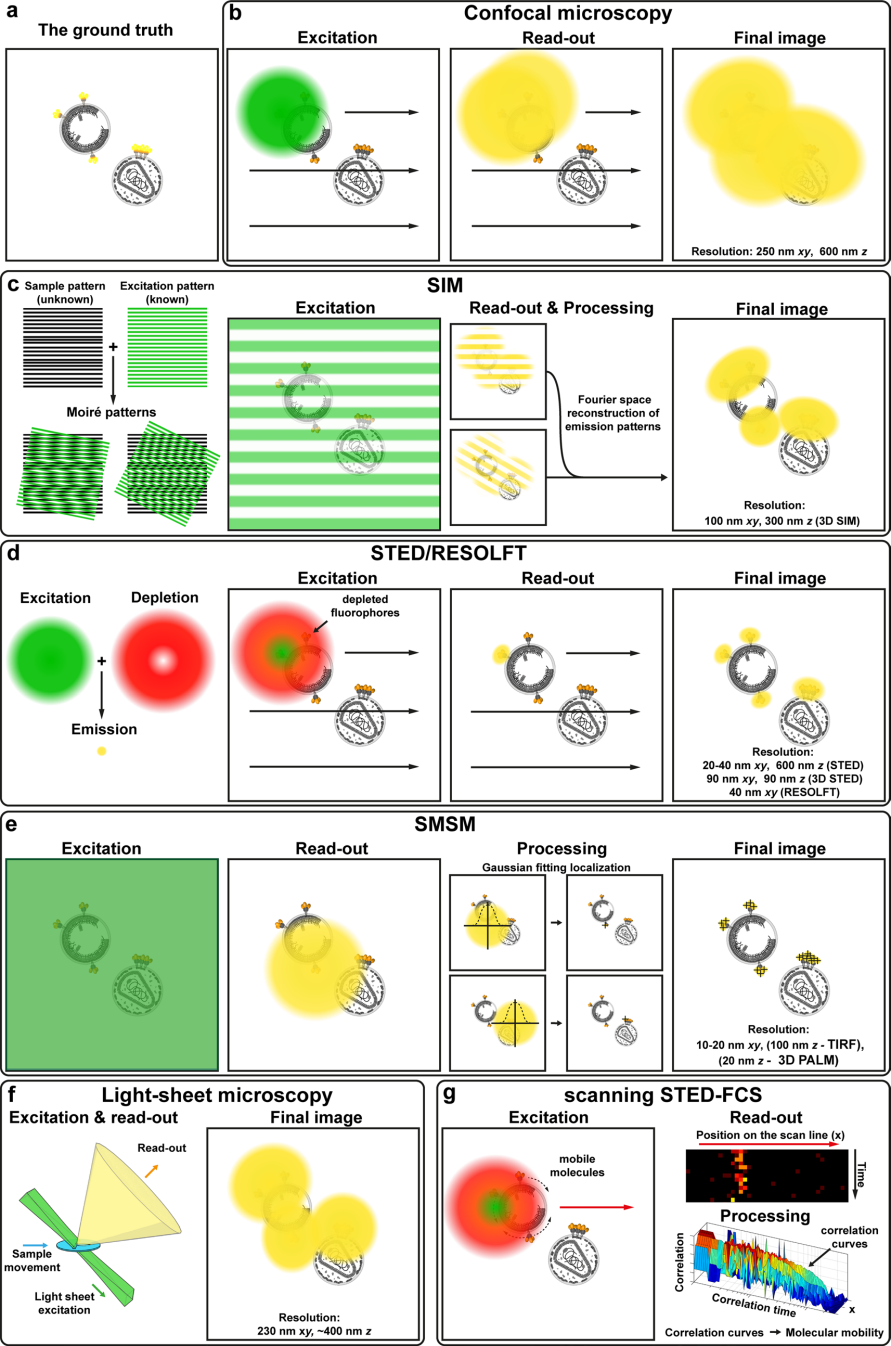
Principles of different super-resolution fluorescence microscopy methods and a comparison of their resolution capabilities. “Excitation” and “Read-out” panels refer to the fluorophore excitation and signal acquisition at a single point in time as the final image is built either by laser scanning (indicated by the arrows) or wide-field illumination of the imaged field of view. Some microscopy techniques require additional post-processing of the acquired “Read-out” snapshots to build the final image, as indicated by the “Processing” panels. For a detailed explanation of each technique please see the corresponding sections. **a** A hypothetical ground truth image of 140 nm mature and immature virus particles with fluorescently tagged Env molecules. Image depth (*z*) has been ignored for the sake of clarity. **b** A standard confocal microscopy delivering a blurred diffraction-limited resolution image. **c** Structured Illumination Microscopy (SIM) (“SIM and related techniques” section). **d** Stimulated Emission Depletion (STED) and Reversible Saturable Optical Fluorescence Transitions (RESOLFT) microscopy (“STED microscopy” section). **e** Single Molecule Switching Microscopy (SMSM) (“Single molecule switching microscopy (SMSM)” section). **f** Light-sheet microscopy. Please note that this technique by itself does not provide much improvement in the spatial resolution, but it is often combined with other super-resolution microscopy techniques due to the general improvements it brings to the imaging of cellular structures (“Light-sheet microscopy” section). **g** Scanning Stimulated Emission Depletion Fluorescence Correlation Spectroscopy (sSTED-FCS) (“Imaging speed” section)

Multiple SRFM approaches have evolved over the years that offer improved spatial resolution over conventional wide-field or laser scanning confocal microscopes (Fig. 2). Approaches such as structured illumination (SIM) [8], image scanning [9], multifocal structured illumination [10], Airyscan [11], or re-scan [12] microscopy achieve a 1.5–2-fold improvement in resolution (down to 100–150 nm). While these approaches offer distinct advantages such as their straightforward applicability to conventionally prepared samples their modest resolution increase has prevented their widespread use in the virus research, where studied virus structures are even smaller. Instead, to date, most of HIV-1 SRFM studies have utilised techniques such as Stimulated Emission Depletion (STED) microscopy [13] or Photo-Activation Localization Microscopy [(f)PALM] [14, 15] and (direct) Stochastic Optical Reconstruction Microscopy [(d)STORM] [16, 17], that offer spatial resolution below 100 nm and thus enable for the analysis of the details of virus architecture as well as interactions between viruses and cell components during virus replication and spread. In the next sections we will introduce the reader into the principles and some technical details of these SRFM approaches, highlighting their advantages as well as disadvantages.

### SIM and related techniques

As highlighted, SIM and related techniques such as image scanning, multifocal structured illumination, Airyscan, or re-scan microscopy achieve a 1.5–2-fold improvement in spatial resolution compared to conventional optical microscopes (down to 100–150 nm). These approaches usually make use of optical properties of the microscope (such as heterogeneity or patterns in the detected signal) in conjunction with distinct image analyses. For example, SIM takes advantage of Moiré pattern effect (Fig. 2c) to reveal sub-diffraction sized information about the sample structures. This is achieved by illuminating a wide field of the sample with a high frequency striped pattern (Fig. 2c—“Excitation”). This light pattern creates the Moiré pattern interference with structures in the sample (Fig. 2c—“Read-out”). A series of camera images (typically more than 9) is obtained by scanning and rotating the illumination pattern. These raw images, in conjunction with distinct image analysis, are then used to reconstruct the final image containing high-resolution information (Fig. 2c—“Processing” and “Final Image”) [8]. The spatio-temporal resolution, ease-of-use, versatility, and reliability (specifically with respect to possible artefacts from the required image analysis) of this approach have been further increased by operating it with Total Internal Reflection Fluorescence illumination (TIRF), which reduces the excitation in axial *z*-direction to ~ 100 nm above the sample coverslip surface [18]. Other improvements include the use of different illumination patterns such as multiple spots instead of stripes [10], adapting principles principles of SIM to confocal setups (Airyscan or re-scan microscopy) [11, 12] or by introducing control measures on the final reconstructed image [19]. Despite their still limited spatial resolution, these approaches are very versatile, offering 3D and live-cell imaging capability that works well with conventional microscopy fluorophores and FPs. Thus they are ideally suited for studies that would benefit even from a modest resolution increase. Unfortunately (as previously indicated), this only modest resolution increase has prevented the widespread use of these approaches in areas such as virus research, which usually require sub-100 nm resolution.

### Sub-100 nm resolution SRFM approaches

Sub-100 nm resolution SRFM approaches achieve subdiffraction scales by switching the fluorescent labels between bright and dark states with only a small subset of all fluorophores being allowed to fluoresce and thus be individually distinguished at any given moment. Combined with the knowledge of the precise position of these fluorescing molecules, this allows for the generation of an image that is no longer restricted by the light diffraction limit [20]. The main difference between switching-based SRFM techniques relates to how the knowledge of the fluorophore position is generated and they can be put into two groups: 1. Targeted shift of excited fluorophore into the dark state at the fringes of a precisely positioned fluorescence excitation spot. This strategy is employed by STED microscopy [13], as well as the related Reversible Saturable Optical (Fluorescence) Transition (RESOLFT) microscopy [21, 22] variant. 2. Stochastic switching of fluorescing molecules in the entire field of view followed by their precise localisation. Techniques based on this approach [here collectively called Single Molecule Switching Microscopy (SMSM)] include (f)PALM [14, 15] and (d)STORM [16, 17], as well as variants thereof such as Ground State Depletion microscopy followed by Individual Molecule return (GSDIM) [23], Point Accumulation for Imaging in Nanoscale Topography (PAINT) [24], or Super-resolution Optical Fluctuation Imaging (SOFI) [25]. The following sections introduce the principles behind these techniques and highlight their advantages as well as disadvantages.

#### STED microscopy

STED SRFM relies on driving excited fluorophores (i.e. in their fluorescent bright state) back into their dark ground state via a non-destructive process employing stimulated emission using additional laser light. Specifically, laser excitation puts fluorophores into their excited state from where they spontaneously return to the ground state emitting a fluorescence photon that can be registered by the microscope detector. When a red-shifted laser (so-called STED laser) is added it acts on already excited fluorophore inducing the return to the ground non-fluorescent state leading to an efficient fluorescence depletion. By modulating the focal intensity distribution of the STED laser in such a way that it features at least one intensity minimum (e.g. a donut-shaped intensity distribution) fluorescence is depleted everywhere except at the local minimum (Fig. 2d).

This effectively creates a sub-diffraction sized excitation spot, which when scanned across the sample (Fig. 2d—“Excitation”) creates an image with sub-diffraction spatial resolution [13, 26, 27] (Fig. 2d—“Final image”). Since the efficiency of fluorescence depletion scales with the intensity of the STED laser, the size of the effective scanning spot and thus the spatial resolution can be tuned accordingly from diffraction limited (i.e. ~ 200 nm with STED laser off) to in principle unlimited scale (usually < 50–60 nm in cellular imaging) [28, 29]. STED microscopy approach can also provide resolution improvement both in lateral and axial directions with < 100 nm axial resolution demonstrated in biological samples [29–32]. Here, a unique property of STED microscopy is the flexibility in designing an experiment by straightforwardly tuning the spatial resolution along all spatial directions. Another advantage of STED microscopy lies in the ability to create a direct image without the need of post processing thus simplifying the acquisition process and avoiding potential post-processing induced image artefacts. While the requirement for high STED laser intensities (GW cm^−2^) raises concerns over increased photobleaching and phototoxicity, this drawback has been efficiently mitigated through improved sample preparation and image acquisition protocols thus making STED microscopy suitable for live-cell observations [33–38]. Overall, due to its ability to directly acquire super-resolved images STED microscopy is well suited for fast live and fixed imaging studies. On the other hand, due to the high laser powers required for efficient fluorophore depletion this technique may not be suitable for long duration live cell imaging.

RESOLFT microscopy represents a variant of STED microscopy which instead of organic fluorophores employs special reversibly photoswitchable fluorescent labels such as reversibly switchable fluorescent proteins (rsFPs) [20–22]. These labels are switched between a fluorescent/bright and a dark state by light induced conformational changes [39]. In a similar fashion to STED microscopy, RESOLFT is also usually employed on a confocal scanning microscope, where switching to the dark state is only induced at the focal periphery using a laser spot with a local intensity zero (such as a donut-shaped intensity distribution) (Fig. 2d). Because switching between the different conformational states requires low laser intensities (~ 1 kW cm^−2^), RESOLFT has been shown to be well suited for live-cell imaging [21, 40], further improved through optimized image acquisition protocols [41–43]. Although the requirement to use special reversible photoswitchable labels can be considered a drawback for this technique, there are already multiple label variants available in several colours [21, 41, 42] and suitable photoswitchable organic dyes are currently in development [44–46].

#### Single molecule switching microscopy (SMSM)

SMSM-based approaches are usually based on wide-field illumination in combination with camera detection (Fig. 2e—“Excitation”). They rely on building a sub-diffraction image from a cycle of 100–10,000 s of individual camera frames where only small subsets of individual isolated fluorescent labels are stochastically switched-on, i.e. allowed in their bright on-state, and a different subset of individual labels is on for each subsequent camera frame (Fig. 2e—“Read-out”). The spatial positions of the individual fluorescing molecules are precisely determined from their recorded blurred fluorescence spots, and positions of all individual labels across all camera frames are then used to construct the final super-resolved image (Fig. 2e—“Processing” and “Final image”). Stochastic on–off switching of single fluorophores is achieved via different means. For example, PALM employs light-induced fluorescence activation of photoactivable fluorescent labels and subsequent photobleaching [15] whereas STORM originally utilised stochastic fluorescence transitions of organic dye pairs [16]. STORM experiments have been further simplified by image acquisition through photoswitching of a single dye only, for example in dSTORM [17] and GSDIM [23]. Finally, photoswitching in PAINT is achieved by excitation of only fluorophores that transiently bind to the membranes of interest either directly [24] or via specific DNA-target detection (DNA-PAINT) [47]. SMSM techniques usually offer a very high resolution enhancement, often achieving 10-20 nm localisation precisions, using relatively simple optical setups. To reduce out-of-focus light and thus optimize single-molecule localisation SMSM is commonly paired with Total Internal Reflection Fluorescence illumination (TIRF) that reduces the excitation in *z*-direction to ~ 100 nm above the sample coverslip surface. SMSM-based imaging has been further improved by optimisations in single-molecule photoswitching conditions [17, 48–50], multi-color imaging [51–53] and introduction of various 3D SMSM modes [54–57]. While current SMSM approaches offer a superior image resolution, a limitation of this technique lies in the requirement for acquisition of many camera frames followed by an extensive image post-processing to create a final super-resolved image. These steps may be a source of bias such as due to the imperfect photoswitching or labelling (see for example [58]) which may cause incomplete visualisation of observed structures when they are present in a low number. The need for longer acquisition times also reduces the time resolution and thus applicability to resolve live-cell dynamics. However, this issue is mitigated by the use of optimised image acquisition and processing protocols [59–63]. In summary, SMSM currently offers the best resolution enhancement out of all popular super-resolution techniques. However, this comes at the cost of several second-long acquisition times thus making this technique less suitable for live cell imaging but very useful for fixed sample studies that require highest possible, molecular level resolution.

### Light-sheet microscopy

While light-sheet microscopy does not per-se supply any improved spatial resolution (Fig. 2f—“Final image”) it is mentioned here due to the general improvements it brings to the imaging of cellular structures. In light-sheet microscopy the sample is illuminated by a beam of light in a shape of a flat plane that is usually generated perpendicularly to the optical axis of the detection objective (Fig. 2f–“Excitation and read-out”). In this approach the fluorescence image of a sample is generated as it moves across the thin area illuminated by the light-sheet [64–66]. This technique offers several advantages over standard fluorescence microscopy approaches which include: (1) Decreased photodamage and phototoxicity as only a small portion of the sample is illuminated at any given time; (2) Increased sample depth penetration due to the perpendicular angle of the illuminating light-sheet; (3) High imaging speed as the sample is illuminated by a plane of light rather than a point source (as is the case in confocal laser scanning microscopy); and (4) Improved signal-to-background ratios due to improved rejection of out-of-focus signals. These advantages make this microscopy technique an excellent tool for live-cell imaging. However, as highlighted, light-sheet microscopy does not offer an increased spatial resolution over conventional microscopes. Approaches such as Bessel beam light-sheet can reduce the thickness of the illumination plane further but this only results in the improvement to the axial resolution [67, 68]. Therefore, for increased lateral resolution, researchers have started to combine light-sheet microscopy with SRFM approaches, such as with SMSM [69] and SIM [70, 71]. Thanks to its advantages light-sheet microscopy is very well suited for live cell imaging studies that require fast acquisitions of large three-dimensional data sets.

## Challenges of SRFM in live cell imaging studies

To date, most of to-date HIV-1 SRFM studies have focussed on the analysis of fixed samples. On the other hand, one of the main advantages of fluorescence microscopy and hence SRFM lies in their potential for live-cell imaging studies. However, while all SRFM approaches can be used to observe live fluorescently labelled samples, the choice of the most suitable technique for virology studies in live conditions must consider not only their resolution capabilities but also imaging speed, sample depth penetration, photobleaching and phototoxicity, as well as accurate labelling.

### Imaging speed

Imaging speed is critical for acquisition of dynamic events in cells and viruses. While SMSM techniques offer a very high spatial resolution this comes at a cost of imaging speed as thousands of photoswitching cycles are required to build up the final image. Although with improvements in hardware and localisation algorithms [59–63] the time resolution has been improved to 0.5–2 s (albeit at the cost of reduced spatial resolution) it might still not be optimal for the live imaging of molecular details of virus-cell interactions. This is because processes such as molecular diffusion and clustering dynamics typically occur within milliseconds at the nanometre scales. Similarly, to SMSM techniques, SIM imaging speed is limited by the time required to acquire fluorescent signal from multiple illumination pattern configurations. While a single-color 2D image of a cell can be acquired at 0.1–1 s resolution [72] this may still be non-ideal for live-cell imaging of fast dynamic processes.

Imaging speeds are faster in STED microscopy. As a laser scanning technique its imaging speed chiefly depends on the imaged field of view i.e. the smaller image, the faster the acquisition. STED-microscopy based studies of HIV-1 uptake into HeLa cells have demonstrated a maximum temporal resolution of 5–10 ms, when employing ultrafast beam-scanners on small regions of interest [73]. On the other hand, parallelized scanning approaches have also been developed to increase imaging speed in large fields of view [74–76].

The temporal resolution can be further increased by combing SRFM with single-molecule-based spectroscopic tools such as single-particle tracking (SPT) or fluorescence correlation spectroscopy (FCS). For example, combining SPT with the principle of photoswitching [77] such as in spt-PALM enabled the single-molecule based monitoring of molecular diffusion patterns of HIV-1 Gag and tsO45 proteins from vesicular stomatitis virus G (VSVG) [78]. On the other hand, FCS measurements enable for the determination of not only molecular mobility but also anomalies in diffusion [79, 80]. This is achieved by recording of the fluorescence signal over time as tagged molecules diffuse in and out of the observation spot. The correlation of these fluctuations is then used to determine the molecular transit times of molecules through the observation area and allows calculation of a value of the diffusion coefficient (Fig. 2g—“Processing”). When combined with STED microscope, (STED–)FCS enables for the determination of molecular diffusion modes of individual molecules with high spatial and temporal resolution [81]. In combination with fast line-scanning, STED-FCS [or scanning STED-FCS (sSTED-FCS)] allows for the observation of multiple positions at once (Fig. 2g—“Excitation and read-out”) and has been applied to study molecular trapping sites at 80-nm spatial resolution in the plasma membrane of living cells [82, 83]. sSTED-FCS has recently been utilised to determine the molecular mobility of proteins on the surface of individual HIV-1 particles [84] as well as molecular dynamics in the interior of the live HeLa and CHO cells [85]. In summary, this technique has high potential for studies of molecular interaction dynamics at cell surfaces such as at virus assembly and fusion sites.

### Sample depth penetration

Sample depth penetration in fluorescence microscopy imaging is generally limited by light scattering and optical aberrations due to refractive index mismatches. This leads to deterioration of image resolution and contrast as well as reduction of signal-to-noise levels, especially in SFRM [86, 87]. Such deteriorating effects can, for example, be addressed through 2-photon-based excitation to reduce scattering [88–90] or the use of microscope objective lenses with a better matching of the sample’s refractive index (such as a glycerol-immersion objective) [86]. Ultimately, this issue is solved by the use of adaptive optics to reduce bias from optical aberrations [91], which has already been shown to significantly improve image quality and resolution in STED microscopy [87].

### Photobleaching and phototoxicity

Laser light exposure, especially at high laser intensities, may lead to the generation of reactive species (such as radicals or singlet oxygen) that cause photobleaching and phototoxicity in living systems resulting in cell death. Consequently, these deteriorating effects have to be considered in any (especially live) fluorescence imaging experiments, thus also in SFRM: (1) SIM: Photobleaching and phototoxicity becomes an issue through the requirement of recording multiple raw images for one final image. This limitation is mitigated by optimization of the optical path and illumination scheme, enabling live-cell recordings even in 3D (for a review see [92]) (2) SMSM: Despite the use of low illumination intensities (kW cm^−2^), the UV laser irradiation often required for photoswitching is a cause of pronounced phototoxicity. This can be minimized through far-red illumination schemes (> 640 nm) or minimization of activation light through the application of distinct labels and buffers (for an overview see [93]). (3) STED/RESOLFT microscopy: STED microscopy typically utilises high-intensity (GW cm^−2^) laser light that may lead to phototoxic effects. On the other hand, optimized sample preparation protocols, fast beam-scanning and the adaptation of the wavelength of the STED-laser have proven STED microscopy as viable tool for live-cell investigations, even when employing fluorescent proteins [33, 34]. Moreover, the aforementioned tunability of the STED microscope enables weighing spatial resolution against high laser intensity (for a review see [4]). RESOLFT microscopy uses much lower laser intensities than STED microscopy, but photobleaching or phototoxicity may still be a problem due to the usually employed near-UV laser light and imperfect photoswitching efficiency of fluorescent labels [94]. Nevertheless, live-cell RESOLFT microscopy has successfully been performed using fast, repetitive, parallelized and/or optimized image acquisition schemes [40, 43].

### Labelling

In general with all SRFM approaches greater care has to be taken with respect to labelling and sample preparations as well as data acquisition and analysis approaches, since the increased resolution of SFRM also enhances sensitivity to artefacts such as background staining or stressed cells. While certain imperfections might be forgiven in conventional microscopy, they are usually not in SRFM [4]. Furthermore, a great care has to be taken when using larger fluorescent tags such as antibodies (as employed in immunolabelling), since spatial resolutions of < 20–30 nm are achieved in some SRFM experiments. Consequently, the size of the tags start to bias the image and thus the determination of the spatial position and organization of the tagged molecules. This caveat makes the use of smaller tags such as nanobodies or click chemistry necessary in SRFM studies (for an overview see [5]).

Live-cell SRFM studies of HIV-1 face further unique issues associated with labelling of virus components with technique compatible fluorophores while maintaining a minimal effect on virus morphology and functions. Although convenient, fluorescence tagging via antibodies or nanobodies has only a limited usability in live-cell imaging since it restricts studies to virus or cell external surfaces only. However, effective strategies based on fluorescence proteins have already been developed for HIV-1 studies via conventional microscopy [95–97] and these can be adopted for live-cell SRFM. Organic-dye compatible HIV-1 tagging strategies via non-fluorescent tags such as tetracysteine (TC) tag [98], SNAP-tag [99], CLIP-tag [100] or artificial amino acids and click chemistry [101] can also offer a viable strategies for conducting live-cell SRFM studies of virus replication cycle. For an in-depth review of HIV-1 fluorescent labelling strategies please refer to the work by Sakin et al. [102].

## SFRM studies of HIV-1

**Fig. 3:**
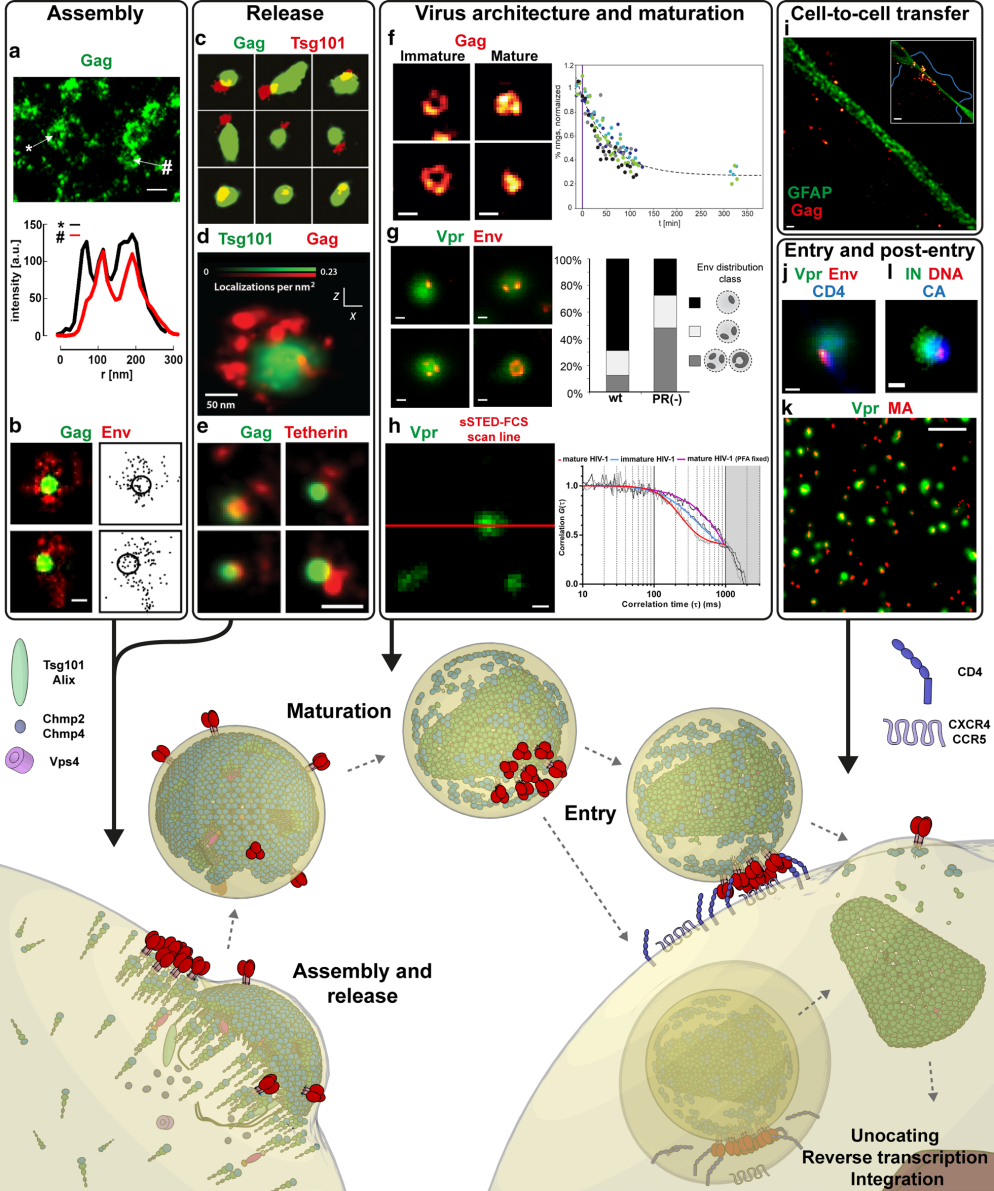
Super-resolution fluorescence microscopy studies and their contribution to the understanding of HIV-1 replication cycle (illustrated in the lower panel). Virus Assembly: **a** dSTORM imaging of cell surface Gag distribution (green) showing representative virus-sized clusters (upper panel) and their fluorescence intensity line profiles (lower panel). Scale bar: 200 nm [105]. The density distributions of Gag protein localizations was found to be similar to the ring-like arrangement of Gag found in immature virus (see panel **f**). **b** dSTORM imaging of Env distribution (red) around cell surface Gag clusters (green). Env molecules (right panels—dots) appear to be largely excluded from the sites of Gag assembly (right panels—circle). Scale bar: 100 nm [109]. Release: **c.** Distribution of Gag (green) and ESCRT protein Tsg101 (red) within budding viruses imaged by dSTORM. Protein localization densities indicate the accumulation of ESCRT proteins at the neck of the virus buds [122]. **d** Distribution of Gag (red) and ESCRT protein Tsg101 (green) within a budding virus imaged by 3D PALM. In this study protein localization densities indicate the existence of ESCRT components within the virus particle. Scale bar: 50 nm [104]. **e** Tetherin clusters (red) at Gag assembly sites (green) imaged by dSTORM Scale bar: 200 nm [108]. Virus architecture and maturation: **f** STED imaging of Gag distribution (red) in immature and mature virus particles showing a 2D projection of ring-like Gag lattice in immature and a central condensed accumulation in mature virus particles (left panels). HIV-1 maturation kinetics was estimated by time-lapse imaging of Gag structures and quantifying the percentage of HIV-1 particles with ring-like distributions over time (right panel). Scale bar: 100 nm [100]. **g** STED imaging of Env distribution (red) on individual eGFP.Vpr tagged virus particles (green) with multi-clustered Env distribution in immature non-infectious particles (PR-) coalescing into a single cluster in mature fully infectious virus (wt) (right panel). Scale bar: 100 nm [112]. **h** sSTED-FCS measurements of Env mobility on individual mature and immature virus particles by fast line-scanning (red line) over individual eGFP.Vpr tagged virus particles (green) and determination of diffusion characteristics at each line pixel using FCS. Representative FCS correlation curve data for Env in mature (red), immature (blue) and fixed (purple) viruses with faster decay indicating increased mobility (right panel). Env was found to undergo maturation-induced increase in mobility indicating its diffusion as one of the causes for Env clustering. Scale bar: 200 nm [84]. Cell-to-cell transfer: **i** Visualising individual virus positions (red/yellow, identified by Gag) by STED microscopy at the contact sites between the infected macrophages (blue cell border in inset) and astrocytes (labelled via glial fibrillary acidic protein (GFAP), green) Scale bar: 500 nm. Inset scale bar: 3 µm [133]. Entry and post-entry: **j.** STED imaging of Env (red) and CD4 (blue) distributions in cell-attached eGFP.Vpr labelled HIV-1 (green) showing a single contact point between Env and CD4. Scale bar: 100 nm [112]. **k** dSTORM image of MA clusters (red) and eGFP.Vpr labelled viruses (green) after their attachment to cells. MA cluster sizes were found to be larger than those in cell-free virus particles. Scale bar: 2 µm [136]. **l** PALM/dSTORM image of RTC/PIC [viral DNA (red), CA (blue) and IN (green)] in the cytoplasm of infected macrophage. Scale bar: 100 nm [138]. Images were modified from indicated references with permission

While SRFM technologies outlined above undergo constant development their application has already provided many novel insights into the previously unexplored details of HIV-1 replication cycle (Fig. 3). The following sections outline how these studies have contributed to the knowledge of HIV-1 replication taking the assembly of a new virus particle as a starting point.

### Assembly

HIV-1 assembles initially as immature particles on the plasma membrane of the infected cells [103]. This process is driven by the virus structural polyprotein Gag as it binds to the inner leaflet of the plasma membrane via matrix (MA) domain and forms hexametric protein shell bound by intermolecular interactions of capsid (CA) domain. Gag is also responsible for the recruitment of other virus and host cell components to the budding site. These include genomic RNA, Gag-Pol polyprotein which also encodes viral enzymes, fusion glycoprotein Env, Viral protein R (Vpr) as well as components of endosomal sorting complex required for transport (ESCRT) machinery [104], which are needed for HIV-1 release. Virus assembly has been very extensively studied via a variety of methods including electron microscopy (EM) and conventional fluorescence microscopy [103]. Well characterised nature of Gag mediated HIV-1 assembly made it a good candidate for a proof-of-concept study of PALM conducted by Betzig and co-workers. This and subsequent SRFM studies [14, 78, 99, 105–109] revealed an existence of 100–200 nm Gag clusters on the surface of COS-7, 293T, HeLa and A3.01 cells. Moreover, a quantitative PALM study has shown that tagged Gag protein clusters are indistinguishable from the mixed clusters of tagged and unmodified Gag suggesting that they represent a true virus assembly sites in the context of Gag transfected COS-7 cells [110]. Finally, 15 nm resolution obtained in a dSTORM study allowed for a visualisation of a ring-like Gag distribution representing a 2D projection of a semi-spherical structure of the HIV-1 Gag shell in A3.01 T cell line (Fig. 3a) [105]. A similar Gag distribution has also been obtained in SRFM imaging of immature virus particle (see “Maturation” section).

In addition to just confirming Gag assembly models derived from previous EM-based studies, SRFM experiments also described novel aspects of Gag assembly inaccessible to conventional light microscopy or EM. Specifically, a spt-PALM live-cell study tracked individual Gag molecules on the plasma membrane of COS-7 cells and demonstrated an existence of two distinct Gag populations; a larger immobile pool of Gag clusters representing virus assembly sites and a more mobile population of individual Gag molecules [78]. Two Gag populations forming either large (at the virus assembly site) or small clusters were also observed in A3.01 cells by a study utilising dSTORM [105]. Finally, the analysis of Gag clusters by quantitative PALM demonstrated that small (< 100 Gag molecules) clusters comprise ~ 40% of all detected Gag clusters suggesting that transition from small-sized clusters to a growing assembly site may represent a rate-limiting step for HIV-1 particle formation in Gag-transfected COS-7 cells [107].

Multi-colour SRFM provides a possibility to study spatial and temporal relationships between virus and cell proteins recruited to the individual virus assembly sites. One of the targets of interest is the fusion glycoprotein Env. Env traffics to the plasma membrane separately from Gag and becomes incorporated into assembled virus via interactions of MA domain of Gag and the cytoplasmic tail of Env (EnvCT) [111]. However, the exact mechanism of incorporation into the virus particle remains unknown. SRFM imaging using (d)STORM in fixed samples has determined Env distribution in the proximity of Gag assembly sites. It demonstrated the existence of large Env clusters at Gag assembly sites in HeLa cells transfected with replication incompetent pCHIV construct that expresses all HIV-1 proteins except Nef (Fig. 3b) [109]. Interestingly, the majority of Env molecules did not colocalize with the assembly site itself but rather was observed in its immediate vicinity. These findings are consistent with 7–10 Env molecules observed via EM and SRFM in budded HIV-1 particles [112, 113]. Furthermore they suggest that, rather than by random incorporation, Env is recruited to the virus budding site via mechanisms that may involve factors other than direct Gag-Env interactions to exclude most of Env from the nascent virus particle [109]. Dynamics of Env incorporation have been studied by Fluorescence Recovery After Photobleaching (FRAP) indicating that Env molecules are immobile in the areas corresponding to Gag structures [114]. Here, SRFM approaches will also be beneficial for, for example, studying the dynamics of Env or Gag at individual (sub-diffraction sized) virus assembly sites. For example, sSTED-FCS was recently used to study diffusion properties of Env on individual virus particles and in the plasma membrane of Env-transfected HeLa cells [84].

Another so far unexplored aspect of virus assembly is the behaviour of lipids at the individual virus assembly sites. Lipidome studies of virus particles revealed a modified lipid content compared to the host-cell membrane, especially an enrichment in saturated lipids, sphingolipids and cholesterol [115–117], indicating sorting of lipids and proteins at the virus assembly site, i.e. viruses potentially arise from so-called “lipid rafts” [118]. However, the exact lipid distribution and their dynamics as Gag assembles on the plasma membrane remain unclear. Experiments with cholera toxin capped GM1 via dSTORM have shown that GM1 does not colocalize with Gag assembly sites in fixed HeLa cells [108], and details of lipid dynamics at individual HIV-1 assembly sites are currently under investigation via (s)STED-FCS.

### Release

The ESCRT machinery is responsible for mediating intracellular fission events such as cytokinesis and the formation of multi-vesicular bodies. HIV-1 hijacks elements of this machinery in order to separate (or bud off) from the plasma membrane of the infected cell [103, 104]. This is achieved by recruiting ESCRT proteins Tsg101 and Alix via p6 domain of Gag which, in turn, recruit further proteins such as Chmp2, Chmp4 and Vps4. While the conventional microscopy studies have provided invaluable insights into the kinetics of the recruitment of ESCRT proteins [119, 120], SRFM has enabled for a closer look at the distribution of ESCRT components within the individual virus budding site. A SMSM-based analysis of ESCRT proteins at the plasma membrane of Gag producing HeLa cells revealed an accumulation of ESCRT proteins in areas of 45–60 nm in diameter at the neck of the budding virus (Fig. 3c). This observation supports a model in which ESCRT proteins accumulate at the plasma membrane below the neck to mediate the scission of the budding virus [121, 122]. However, these observations are inconsistent with the results of a 3D-PALM study in COS-7 cells which indicated that the ESCRT protein machinery accumulated at the head of the budding virus. This study supports a different model where ESCRT filaments grow away from the viral head towards the plasma membrane to mediate scission (Fig. 3d) [123].

SRFM-based experiments have also provided new details of the tetherin (CD317)-mediated restriction of HIV-1 release restriction. Tetherin prevents HIV-1 release from the cell surface by forming a physical link between the budded virus and the plasma membrane [124, 125]. This tetherin-mediated restriction pathway is counteracted by the viral protein U (Vpu), which removes tetherin from the plasma membrane. Quantitative dSTORM analysis of tetherin at Vpu-negative virus assembly sites in HeLa cells highlighted that each site contained 4-7 theterin dimers (Fig. 3e) [108].

### Maturation

Concurrently with virus budding, the viral protease (PR) cleaves the Gag protein lattice inside the virus in a series of tightly regulated steps to release individual proteins, namely MA (matrix), CA (capsid), NC (nucleocapsid) and p6. This PR activity reorganises the virus architecture from an immature and non-infectious into a mature and fully infectious form, characterised by a conical capsid (Fig. 1). This process is termed maturation and it is a critical step in HIV-1 replication cycle as it primes newly produced virus particles for infection of other cells. The architecture of both mature and immature virus particles has been extensively studied by EM based approaches [103]. However, with the ability for the determination of the relative distribution of fluorescently tagged viral proteins and studying dynamic properties of maturation, SRFM-based studies have contributed with essential novel insights into this stage of virus replication.

SRFM has been used to study the distribution and dynamics of virus internal structures during maturation. A PALM study introduced an approach for discriminating between VSV-G pseudotyped mature and immature HIV-1 particles. It relies on the statistical analysis of the signal intensity distributions detected from labelled integrase enzyme domain (IN) of Gag-Pol polyprotein [126]. Immature viruses displayed compact clusters, while mature viruses were characterized by more elongated spots, which were interpreted as conical viral capsids. However, dynamic analysis of virus maturation was infeasible in this study due to the insufficient temporal resolution of the PALM experiments. This limitation was addressed in a STED microscopy study of virus maturation, where the semi-spherical Gag lattice of the immature replication incompetent pCHIV virus particle was visualized and accurately distinguished from the condensed protein distribution found in fully mature virus. The use of a photodestructible viral protease inhibitor enabled for the synchronisation of the virus maturation process, allowing for time-resolved observations of the disassembly of Gag lattice (Fig. 3f) [100]. A detailed analysis of the data revealed a maturation kinetics half-time of ~ 30 min and demonstrated that proteolysis directly induces morphological conversion without further delay, thus making it a rate-limiting step in HIV-1 maturation. This study represented the first time-lapse visualisation of maturation induced reorganisations within individual HIV-1 particles.

Previous EM-based study has suggested an irregular Env distribution on virus surface [113]. Thanks to higher throughput and specific labelling, STED microscopy based experiments allowed for imaging of Env distribution on a large number of individual virus particles generated from 293T cells transfected with replication incompetent pCHIV construct [112]. Env distribution analysis revealed that the surface of immature HIV-1 particles is characterized by multiple separated Env molecules while that of mature particles by only a single Env cluster (Fig. 3g) [112]. This study thus demonstrated the existence of a novel “inside-out” mechanism where PR-induced disassembly of the Gag lattice inside the virus and allows for multi-clustered Env to coalesce into a single cluster in fully infectious mature particles. As multiple Env trimers are required for virus fusion [127, 128] this mechanism ensures that immature virus particles with a broad distribution of single Env molecules are unable to fuse with the target cell membrane until the virus reaches morphological maturity with multiple Env molecules gathered into a single cluster.

The above study has suggested that Gag lattice disassembly mediated clustering of Env molecules may result from an increase in Env mobility upon maturation. Measurements of Env molecular mobility on the surface of individual virus particles via sSTED-FCS have confirmed that Env mobility is dependent on the virus maturation status in pCHIV particles (Fig. 3h) [84]. This study has also demonstrated that the virus surface is generally a very low-mobility environment, where protein mobility is two orders of magnitude slower than on the plasma member of the cell. This is thought to be mainly due to the highly packed lipid environment stemming from the large portion of saturated lipids in the viral membrane [84, 115, 116]. These sSTED-FCS measurements provided, for the first time, information on the dynamic properties of molecules within subdiffraction sized highly curved virus envelopes.

### Spread and persistence

HIV-1 has evolved many mechanisms to facilitate efficient spread and persistence in the infected hosts. These mechanisms include direct cell-to-cell transfer via virological synapses [129], establishment of virus reservoirs [130], and the modulation of the infected cell via HIV accessory proteins [131]. However, to date, only few SRFM studies have targeted these aspects of HIV-1 infection. For example, SRFM was used to track the position of HIV-1 accessory protein Nef in transfected HeLa cells. Nef promotes HIV-1 immune evasion by downregulating immune signalling molecules such as MHC-I in infected cells. Here, SRFM was used for high precision localisation of Nef/MHC-I complexes in individual early and late endosome vesicles as well as in the Trans-Golgi network [132]. A recent STED microscopy study also tracked the trapping and sequestration of fully infectious macrophage produced virus particles inside astrocytes (Fig. 3i) [133]. Astrocytes are one of the HIV-1 reservoirs in the brain but it is currently unclear whether these cells support virus replication or only act as passive HIV-1 reservoirs. The multicolour STED analysis of individual HIV-1 particles sequestered inside astrocytes highlighted that they do not fuse with the astrocytes plasma membrane and therefore do not infect them [133]. Rather, astrocytes act only as a passive reservoir of HIV-1 particles. While challenging, SRFM studies of cell-to-cell transmission and virus reservoirs have the potential to provide novel details on the distribution and dynamics of the molecules involved thus contributing to the analysis of HIV-1 replication and spread.

### Virus entry

HIV-1 entry into the target cell is mediated by binding of Env to cell surface or endocytosed CD4 receptors and CXCR4/CCR5 chemokine co-receptors. Attachment of individual pCHIV particles to cluster of CD4 receptors has been observed via STED microscopy in SupT1R5 cells [112]. Images have shown single clusters of Env oriented towards CD4 clusters on the cell surface indicating direct interactions between Env and CD4 clusters (Fig. 3j). This study has also demonstrated that the cell contact can induce reclustering of mobile Env molecules on the virus surface, presumably through progressive capture of individual Env trimers by virus facing CD4 molecules. These findings are in agreement with cryo-ET studies that proposed the existence of an “entry claw” structure that connects viruses and cell membranes [134]. In another application of SRFM to HIV-1 entry studies, 3D STORM imaging was used for high resolution visualisation of the exposure of neutralizing and non-neutralizing epitopes on single HIV-1_JRFL_ pseudoviruses bound to TZM-bl CD4 T cells [135].

SRFM has also been used to study possible rearrangements of virus internal proteins during attachment and entry. A dSTORM study has visualised the distribution of MA and CA proteins in unbound and cell-attached fully infectious virus particles, highlighting an increase in cluster sizes of MA and CA in virus particles after cellular internalization, suggesting that virus internal structures undergo rearrangments during the entry (Fig. 3k) [136]. A subsequent study using a combination of EM and SRFM imaging indicated that the reported increase in size of mature HIV-1 particles is solely triggered by CD4-Env attachment and therefore it is independent of virus fusion [137]. The observed virus expansion may thus be a manifestation of a novel mechanism that primes HIV-1 for fusion. However, it is currently unclear what virus-intrinsic mechanism may be responsible for the remodelling of the virus envelope membrane, which would be required for such an event.

### Post-entry events

Following entry of the HIV-1 capsid into the cell cytoplasm, the virus genomic RNA is transcribed into double stranded DNA by reverse transcriptase (RT) and integrated into the cellular genome by the viral integrase (IN). The so called reverse transcription complex (RTC) and pre-integration complex (PIC), which are comprised of viral genome and proteins, facilitate reverse transcription, trafficking and nuclear import. Despite the fact that the subdiffraction size and transient occurrence of these complexes makes an analysis of their structural details and dynamics well suited for SRFM studies, these post-entry events are still the least understood phase of the virus replication cycle. Nevertheless, PALM was already used to compare the architecture of fluorescent IN labelled structures in VSV-G pseudotyped cell free virions and post entry HIV-1 subviral complexes [126]. Analysis of the spatial distribution of labelled IN revealed that structures resembling IN complex are mainly present in the cell cytoplasm with only smaller IN structures detected in the cell nucleus. This study has also reported the presence of CA molecules in these cytoplasmic complexes. This result is consistent with findings of another dSTORM/PALM study that visualised fully infectious HIV-1-derived proteins in dsDNA containing post entry RTC/PIC complexes (Fig. 3l). Here, CA was also found in cytoplasmic RTC/PICs and in nuclear PICs, but only in primary human macrophages and not in HeLa cells [138]. The presence of CA was also detected in nuclear PICs of CHO cells imaged by SIM [139]. These findings suggest that there are host cell dependent differences in the degree of capsid disassembly as HIV-1 post-entry complexes travel towards the nucleus.

## Conclusions

Since their introduction, SRFM methods have now reached a high state of maturity, and with the increasing availability of commercial turn-key systems they have the potential to become a standard approach for bioimaging. However, it is clear that there is no one-fits-all approach, and as highlighted each technique comes with a unique set of advantages and disadvantages. On top of that SRFM technology is continuously evolving, with the refinement of existing techniques and combinatorial approaches allowing to mitigate disadvantages of each technique.

Virus research with its clear reason to look beyond the diffraction barrier took an early advantage of this field, and SRFM studies have already provided many novel insights into the understating of the HIV-1 replication cycle. Yet, arguably these are still early days of SRFM imaging with many more aspects of HIV-1 that still await investigation. Moreover, to date most of SRFM HIV-1 studies have been performed in the context of fixed viruses and in vitro cell cultures. On the other hand, SRFM approaches are particularly suitable to study the dynamic behaviour of individual subviral structures and their interactions with cell components in the context of live cells or tissues, and it is in this area where they hold the most potential for future improvements in the understanding of virus replication cycle.

## Abbreviations

FM: Fluorescence microscopy
EM: Electron microscopy
cryo-ET: cryo electron tomography
SRFM: Super-resolution fluorescence microscopy
HIV-1: Human immunodeficiency virus type 1
SIM: Structured illumination microscopy
STED: Stimulated emission depletion microscopy
RESOLFT: Reversible saturable optical fluorescence transition microscopy
SMSM: Single molecule switching microscopy
PALM: Photo-activation localization microscopy
(d)STORM: (direct) Stochastic optical reconstruction microscopy
GSDIM: Ground state depletion microscopy followed by individual molecule return
PAINT: Point accumulation for imaging in nanoscale topography
MINFLUX: Minimal emission fluxes microscopy
TIRF: Total internal reflection fluorescence
SPT: Single particle tracking
FCS: Fluorescence Correlation spectroscopy
sSTED-FCS: Scanning stimulated emission depletion fluorescence correlation spectroscopy
MA: Matrix
CA: Capsid
NC: Nucelocapsid
PR: Protease
RT: Reverse transcriptase
IN: Integrase
ESCRT: Endosomal sorting complex required for transport
FRAP: Fluorescence recovery after photobleaching
GFAP: Glial fibrillary acidic protein

## References

[1] Abbe E (1873). Beiträge zur Theorie des Mikroskops und der mikroskopischen Wahrnehmung. Arch Für Mikrosk Anat.

[2] Nieuwenhuizen RPJ, Lidke KA, Bates M, Puig DL, Grünwald D, Stallinga S (2013). Measuring image resolution in optical nanoscopy. Nat Methods.

[3] Mak J, de Marco A (2018). Recent advances in retroviruses via cryo-electron microscopy. Retrovirology.

[4] Eggeling C, Willig KI, Sahl SJ, Hell SW (2015). Lens-based fluorescence nanoscopy. Q Rev Biophys.

[5] Hell SW, Sahl SJ, Bates M, Zhuang X, Heintzmann R, Booth MJ (2015). The 2015 super-resolution microscopy roadmap. J Phys Appl Phys.

[6] Sahl SJ, Hell SW, Jakobs S (2017). Fluorescence nanoscopy in cell biology. Nat Rev Mol Cell Biol.

[7] Balzarotti F, Eilers Y, Gwosch KC, Gynnå AH, Westphal V, Stefani FD (2017). Nanometer resolution imaging and tracking of fluorescent molecules with minimal photon fluxes. Science.

[8] Gustafsson MG (2000). Surpassing the lateral resolution limit by a factor of two using structured illumination microscopy. J Microsc.

[9] Müller CB, Enderlein J (2010). Image scanning microscopy. Phys Rev Lett.

[10] York AG, Parekh SH, Nogare DD, Fischer RS, Temprine K, Mione M (2012). Resolution doubling in live, multicellular organisms via multifocal structured illumination microscopy. Nat Methods.

[11] Korobchevskaya K, Lagerholm B, Colin-York H, Fritzsche M (2017). Exploring the potential of airyscan microscopy for live cell imaging. Photonics.

[12] De Luca GMR, Breedijk RMP, Brandt RAJ, Zeelenberg CHC, de Jong BE, Timmermans W (2013). Re-scan confocal microscopy: scanning twice for better resolution. Biomed Opt Express.

[13] Hell SW, Wichmann J (1994). Breaking the diffraction resolution limit by stimulated emission: stimulated-emission-depletion fluorescence microscopy. Opt Lett.

[14] Betzig E, Patterson GH, Sougrat R, Lindwasser OW, Olenych S, Bonifacino JS (2006). Imaging intracellular fluorescent proteins at nanometer resolution. Science.

[15] Hess ST, Girirajan TPK, Mason MD (2006). Ultra-high resolution imaging by fluorescence photoactivation localization microscopy. Biophys J.

[16] Rust MJ, Bates M, Zhuang X (2006). Sub-diffraction-limit imaging by stochastic optical reconstruction microscopy (STORM). Nat Methods.

[17] Heilemann M, van de Linde S, Schüttpelz M, Kasper R, Seefeldt B, Mukherjee A (2008). Subdiffraction-resolution fluorescence imaging with conventional fluorescent probes. Angew Chem Int Ed.

[18] Li D, Shao L, Chen B-C, Zhang X, Zhang M, Moses B (2015). Extended-resolution structured illumination imaging of endocytic and cytoskeletal dynamics. Science.

[19] Ball G, Demmerle J, Kaufmann R, Davis I, Dobbie IM, Schermelleh L (2015). SIMcheck: a toolbox for successful super-resolution structured illumination microscopy. Sci Rep.

[20] Hell SW, Jakobs S, Kastrup L (2003). Imaging and writing at the nanoscale with focused visible light through saturable optical transitions. Appl Phys Mater Sci Process.

[21] Grotjohann T, Testa I, Leutenegger M, Bock H, Urban NT, Lavoie-Cardinal F (2011). Diffraction-unlimited all-optical imaging and writing with a photochromic GFP. Nature.

[22] Hofmann M, Eggeling C, Jakobs S, Hell SW (2005). Breaking the diffraction barrier in fluorescence microscopy at low light intensities by using reversibly photoswitchable proteins. Proc Natl Acad Sci.

[23] Fölling J, Bossi M, Bock H, Medda R, Wurm CA, Hein B (2008). Fluorescence nanoscopy by ground-state depletion and single-molecule return. Nat Methods.

[24] Sharonov A, Hochstrasser RM (2006). Wide-field subdiffraction imaging by accumulated binding of diffusing probes. Proc Natl Acad Sci.

[25] Dertinger T, Colyer R, Iyer G, Weiss S, Enderlein J (2009). Fast, background-free, 3D super-resolution optical fluctuation imaging (SOFI). Proc Natl Acad Sci.

[26] Klar TA, Jakobs S, Dyba M, Egner A, Hell SW (2000). Fluorescence microscopy with diffraction resolution barrier broken by stimulated emission. Proc Natl Acad Sci USA.

[27] Klar TA, Engel E, Hell SW (2001). Breaking Abbe’s diffraction resolution limit in fluorescence microscopy with stimulated emission depletion beams of various shapes. Phys Rev E.

[28] Westphal V, Hell SW (2005). nanoscale resolution in the focal plane of an optical microscope. Phys Rev Lett.

[29] Harke B, Keller J, Ullal CK, Westphal V, Schönle A, Hell SW (2008). Resolution scaling in STED microscopy. Opt Express.

[30] Osseforth C, Moffitt JR, Schermelleh L, Michaelis J (2014). Simultaneous dual-color 3D STED microscopy. Opt Express.

[31] Schmidt R, Wurm CA, Jakobs S, Engelhardt J, Egner A, Hell SW (2008). Spherical nanosized focal spot unravels the interior of cells. Nat Methods.

[32] Hell SW, Schmidt R, Egner A (2009). Diffraction-unlimited three-dimensional optical nanoscopy with opposing lenses. Nat Photonics.

[33] Bottanelli F, Kromann EB, Allgeyer ES, Erdmann RS, Wood Baguley S, Sirinakis G (2016). Two-colour live-cell nanoscale imaging of intracellular targets. Nat Commun.

[34] Nagerl UV, Willig KI, Hein B, Hell SW, Bonhoeffer T (2008). Live-cell imaging of dendritic spines by STED microscopy. Proc Natl Acad Sci.

[35] Westphal V, Rizzoli SO, Lauterbach MA, Kamin D, Jahn R, Hell SW (2008). Video-rate far-field optical nanoscopy dissects synaptic vesicle movement. Science.

[36] Vicidomini G, Moneron G, Han KY, Westphal V, Ta H, Reuss M (2011). Sharper low-power STED nanoscopy by time gating. Nat Methods.

[37] Staudt T, Engler A, Rittweger E, Harke B, Engelhardt J, Hell SW (2011). Far-field optical nanoscopy with reduced number of state transition cycles. Opt Express.

[38] Heine J, Reuss M, Harke B, D’Este E, Sahl SJ, Hell SW (2017). Adaptive-illumination STED nanoscopy. Proc Natl Acad Sci.

[39] Andresen M, Wahl MC, Stiel AC, Grater F, Schafer LV, Trowitzsch S (2005). Structure and mechanism of the reversible photoswitch of a fluorescent protein. Proc Natl Acad Sci.

[40] Testa I, Urban NT, Jakobs S, Eggeling C, Willig KI, Hell SW (2012). Nanoscopy of living brain slices with low light levels. Neuron.

[41] Lavoie-Cardinal F, Jensen NA, Westphal V, Stiel AC, Chmyrov A, Bierwagen J (2014). Two-color RESOLFT nanoscopy with green and red fluorescent photochromic proteins. ChemPhysChem.

[42] Tiwari DK, Arai Y, Yamanaka M, Matsuda T, Agetsuma M, Nakano M (2015). A fast- and positively photoswitchable fluorescent protein for ultralow-laser-power RESOLFT nanoscopy. Nat Methods.

[43] Masullo L, Boden A, Pennacchietti F, Coceano G, Ratz M, Testa I. Enhanced photon collection enables four dimensional fluorescence nanoscopy of living systems. 2018; Available from http://biorxiv.org/lookup/doi/10.1101/248880. 10.1038/s41467-018-05799-w PMC6095837 30115928

[44] Kwon J, Hwang J, Park J, Han GR, Han KY, Kim SK (2016). RESOLFT nanoscopy with photoswitchable organic fluorophores. Sci Rep.

[45] Roubinet B, Bossi ML, Alt P, Leutenegger M, Shojaei H, Schnorrenberg S (2016). Carboxylated photoswitchable diarylethenes for biolabeling and super-resolution RESOLFT microscopy. Angew Chem Int Ed.

[46] Xiong Y, Vargas Jentzsch A, Osterrieth JWM, Sezgin E, Sazanovich IV, Reglinski K (2018). Spironaphthoxazine switchable dyes for biological imaging. Chem Sci.

[47] Jungmann R, Avendaño MS, Woehrstein JB, Dai M, Shih WM, Yin P (2014). Multiplexed 3D cellular super-resolution imaging with DNA-PAINT and Exchange-PAINT. Nat Methods.

[48] Vogelsang J, Cordes T, Forthmann C, Steinhauer C, Tinnefeld P (2009). Controlling the fluorescence of ordinary oxazine dyes for single-molecule switching and superresolution microscopy. Proc Natl Acad Sci.

[49] Dempsey GT, Vaughan JC, Chen KH, Bates M, Zhuang X (2011). Evaluation of fluorophores for optimal performance in localization-based super-resolution imaging. Nat Methods.

[50] van de Linde S, Endesfelder U, Mukherjee A, Schüttpelz M, Wiebusch G, Wolter S (2009). Multicolor photoswitching microscopy for subdiffraction-resolution fluorescence imaging. Photochem Photobiol Sci.

[51] Shroff H, Galbraith CG, Galbraith JA, White H, Gillette J, Olenych S (2007). Dual-color superresolution imaging of genetically expressed probes within individual adhesion complexes. Proc Natl Acad Sci.

[52] Bock H, Geisler C, Wurm CA, von Middendorff C, Jakobs S, Schönle A (2007). Two-color far-field fluorescence nanoscopy based on photoswitchable emitters. Appl Phys B.

[53] Bates M, Huang B, Dempsey GT, Zhuang X (2007). Multicolor super-resolution imaging with photo-switchable fluorescent probes. Science.

[54] Huang B, Wang W, Bates M, Zhuang X (2008). Three-dimensional super-resolution imaging by stochastic optical reconstruction microscopy. Science.

[55] Juette MF, Gould TJ, Lessard MD, Mlodzianoski MJ, Nagpure BS, Bennett BT (2008). Three-dimensional sub–100 nm resolution fluorescence microscopy of thick samples. Nat Methods.

[56] Shtengel G, Galbraith JA, Galbraith CG, Lippincott-Schwartz J, Gillette JM, Manley S (2009). Interferometric fluorescent super-resolution microscopy resolves 3D cellular ultrastructure. Proc Natl Acad Sci.

[57] Pavani SRP, Thompson MA, Biteen JS, Lord SJ, Liu N, Twieg RJ (2009). Three-dimensional, single-molecule fluorescence imaging beyond the diffraction limit by using a double-helix point spread function. Proc Natl Acad Sci.

[58] Durisic N, Laparra-Cuervo L, Sandoval-Álvarez Á, Borbely JS, Lakadamyali M (2014). Single-molecule evaluation of fluorescent protein photoactivation efficiency using an in vivo nanotemplate. Nat Methods.

[59] Jones SA, Shim S-H, He J, Zhuang X (2011). Fast, three-dimensional super-resolution imaging of live cells. Nat Methods.

[60] Huang F, Hartwich TMP, Rivera-Molina FE, Lin Y, Duim WC, Long JJ (2013). Video-rate nanoscopy using sCMOS camera–specific single-molecule localization algorithms. Nat Methods.

[61] Min J, Vonesch C, Kirshner H, Carlini L, Olivier N, Holden S (2015). FALCON: fast and unbiased reconstruction of high-density super-resolution microscopy data. Sci Rep.

[62] Cox S, Rosten E, Monypenny J, Jovanovic-Talisman T, Burnette DT, Lippincott-Schwartz J (2012). Bayesian localization microscopy reveals nanoscale podosome dynamics. Nat Methods.

[63] Gustafsson N, Culley S, Ashdown G, Owen DM, Pereira PM, Henriques R (2016). Fast live-cell conventional fluorophore nanoscopy with ImageJ through super-resolution radial fluctuations. Nat Commun.

[64] Power RM, Huisken J (2017). A guide to light-sheet fluorescence microscopy for multiscale imaging. Nat Methods.

[65] Voie AH, Burns DH, Spelman FA (1993). Orthogonal-plane fluorescence optical sectioning: three-dimensional imaging of macroscopic biological specimens. J Microsc.

[66] Huisken J (2004). Optical sectioning deep inside live embryos by selective plane illumination microscopy. Science.

[67] Chen B-C, Legant WR, Wang K, Shao L, Milkie DE, Davidson MW (2014). Lattice light-sheet microscopy: Imaging molecules to embryos at high spatiotemporal resolution. Science.

[68] Planchon TA, Gao L, Milkie DE, Davidson MW, Galbraith JA, Galbraith CG (2011). Rapid three-dimensional isotropic imaging of living cells using Bessel beam plane illumination. Nat Methods.

[69] Cella Zanacchi F, Lavagnino Z, Perrone Donnorso M, Del Bue A, Furia L, Faretta M (2011). Live-cell 3D super-resolution imaging in thick biological samples. Nat Methods.

[70] Gao L, Shao L, Higgins CD, Poulton JS, Peifer M, Davidson MW (2012). Noninvasive imaging beyond the diffraction limit of 3D dynamics in thickly fluorescent specimens. Cell.

[71] Chang B-J, Perez Meza VD, Stelzer EHK (2017). csiLSFM combines light-sheet fluorescence microscopy and coherent structured illumination for a lateral resolution below 100 nm. Proc Natl Acad Sci.

[72] Kner P, Chhun BB, Griffis ER, Winoto L, Gustafsson MGL (2009). Super-resolution video microscopy of live cells by structured illumination. Nat Methods.

[73] Schneider J, Zahn J, Maglione M, Sigrist SJ, Marquard J, Chojnacki J (2015). Ultrafast, temporally stochastic STED nanoscopy of millisecond dynamics. Nat Methods.

[74] Schwentker MA, Bock H, Hofmann M, Jakobs S, Bewersdorf J, Eggeling C (2007). Wide-field subdiffraction RESOLFT microscopy using fluorescent protein photoswitching. Microsc Res Tech.

[75] Chmyrov A, Keller J, Grotjohann T, Ratz M, d’Este E, Jakobs S (2013). Nanoscopy with more than 100,000 “doughnuts”. Nat Methods.

[76] Yang B, Przybilla F, Mestre M, Trebbia J-B, Lounis B (2014). Large parallelization of STED nanoscopy using optical lattices. Opt Express.

[77] Eggeling C, Hilbert M, Bock H, Ringemann C, Hofmann M, Stiel AC (2007). Reversible photoswitching enables single-molecule fluorescence fluctuation spectroscopy at high molecular concentration. Microsc Res Tech.

[78] Manley S, Gillette JM, Patterson GH, Shroff H, Hess HF, Betzig E (2008). High-density mapping of single-molecule trajectories with photoactivated localization microscopy. Nat Methods.

[79] Magde D, Elson E, Webb WW (1972). Thermodynamic fluctuations in a reacting system—measurement by fluorescence correlation spectroscopy. Phys Rev Lett.

[80] Schwille P, Korlach J, Webb WW (1999). Fluorescence correlation spectroscopy with single-molecule sensitivity on cell and model membranes. Cytometry.

[81] Eggeling C, Ringemann C, Medda R, Schwarzmann G, Sandhoff K, Polyakova S (2009). Direct observation of the nanoscale dynamics of membrane lipids in a living cell. Nature.

[82] Honigmann A, Mueller V, Ta H, Schoenle A, Sezgin E, Hell SW (2014). Scanning STED-FCS reveals spatiotemporal heterogeneity of lipid interaction in the plasma membrane of living cells. Nat Commun.

[83] Benda A, Ma Y, Gaus K (2015). Self-calibrated line-scan STED-FCS to quantify lipid dynamics in model and cell membranes. Biophys J.

[84] Chojnacki J, Waithe D, Carravilla P, Huarte N, Galiani S, Enderlein J (2017). Envelope glycoprotein mobility on HIV-1 particles depends on the virus maturation state. Nat Commun.

[85] Lanzanò L, Scipioni L, Di Bona M, Bianchini P, Bizzarri R, Cardarelli F (2017). Measurement of nanoscale three-dimensional diffusion in the interior of living cells by STED-FCS. Nat Commun.

[86] Urban NT, Willig KI, Hell SW, Nägerl UV (2011). STED nanoscopy of actin dynamics in synapses deep inside living brain slices. Biophys J.

[87] Gould TJ, Burke D, Bewersdorf J, Booth MJ (2012). Adaptive optics enables 3D STED microscopy in aberrating specimens. Opt Express.

[88] Takasaki KT, Ding JB, Sabatini BL (2013). Live-cell superresolution imaging by pulsed STED two-photon excitation microscopy. Biophys J.

[89] Moneron G, Hell SW (2009). Two-photon excitation STED microscopy. Opt Express.

[90] Fölling J, Belov V, Riedel D, Schönle A, Egner A, Eggeling C (2008). fluorescence nanoscopy with optical sectioning by two-photon induced molecular switching using continuous-wave lasers. ChemPhysChem.

[91] Booth MJ (2014). Adaptive optical microscopy: the ongoing quest for a perfect image. Light Sci Appl.

[92] Heintzmann R, Huser T (2017). Super-resolution structured illumination microscopy. Chem Rev.

[93] Wäldchen S, Lehmann J, Klein T, van de Linde S, Sauer M (2015). Light-induced cell damage in live-cell super-resolution microscopy. Sci Rep.

[94] Xiong Y, Rivera-Fuentes P, Sezgin E, Vargas Jentzsch A, Eggeling C, Anderson HL (2016). Photoswitchable spiropyran dyads for biological imaging. Org Lett.

[95] Hubner W, McNerney GP, Chen P, Dale BM, Gordon RE, Chuang FYS (2009). Quantitative 3D video microscopy of HIV transfer across T cell virological synapses. Science.

[96] Ivanchenko S, Godinez WJ, Lampe M, Kräusslich H-G, Eils R, Rohr K (2009). Dynamics of HIV-1 assembly and release. Mothes W, editor. PLoS Pathog.

[97] Nakane S, Iwamoto A, Matsuda Z (2015). The V4 and V5 variable loops of HIV-1 envelope glycoprotein are tolerant to insertion of green fluorescent protein and are useful targets for labeling. J Biol Chem.

[98] Pereira CF, Ellenberg PC, Jones KL, Fernandez TL, Smyth RP, Hawkes DJ (2011). Labeling of multiple HIV-1 proteins with the biarsenical-tetracysteine system. Aiyar A, editor. PLoS ONE.

[99] Eckhardt M, Anders M, Muranyi W, Heilemann M, Krijnse-Locker J, Müller B (2011). A SNAP-tagged derivative of HIV-1—a versatile tool to study virus-cell interactions. Ambrose Z, editor. PLoS ONE.

[100] Hanne J, Göttfert F, Schimer J, Anders-Össwein M, Konvalinka J, Engelhardt J (2016). Stimulated emission depletion nanoscopy reveals time-course of human immunodeficiency virus proteolytic maturation. ACS Nano.

[101] Sakin V, Hanne J, Dunder J, Anders-Össwein M, Laketa V, Nikić I (2017). A versatile tool for live-cell imaging and super-resolution nanoscopy studies of HIV-1 env distribution and mobility. Cell Chem Biol.

[102] Sakin V, Paci G, Lemke EA, Müller B (2016). Labeling of virus components for advanced, quantitative imaging analyses. FEBS Lett.

[103] Freed EO (2015). HIV-1 assembly, release and maturation. Nat Rev Microbiol.

[104] Lippincott-Schwartz J, Freed EO, van Engelenburg SB (2017). A consensus view of ESCRT-mediated human immunodeficiency virus Type 1 abscission. Annu Rev Virol.

[105] Malkusch S, Muranyi W, Müller B, Kräusslich H-G, Heilemann M (2013). Single-molecule coordinate-based analysis of the morphology of HIV-1 assembly sites with near-molecular spatial resolution. Histochem Cell Biol.

[106] Helma J, Schmidthals K, Lux V, Nüske S, Scholz AM, Kräusslich H-G (2012). Direct and dynamic detection of HIV-1 in living cells. Marcello A, editor. PLoS ONE.

[107] Gunzenhäuser J, Olivier N, Pengo T, Manley S (2012). Quantitative super-resolution imaging reveals protein stoichiometry and nanoscale morphology of assembling HIV-gag virions. Nano Lett.

[108] Lehmann M, Rocha S, Mangeat B, Blanchet F, Uji-i H, Hofkens J (2011). Quantitative multicolor super-resolution microscopy reveals tetherin HIV-1 interaction. Krausslich H-G, editor. PLoS Pathog.

[109] Muranyi W, Malkusch S, Müller B, Heilemann M, Kräusslich H-G (2013). Super-resolution microscopy reveals specific recruitment of HIV-1 envelope proteins to viral assembly sites dependent on the envelope C-terminal tail. Trkola A, editor. PLoS Pathog.

[110] Gunzenhäuser J, Wyss R, Manley S (2014). A quantitative approach to evaluate the impact of fluorescent labeling on membrane-bound HIV-gag assembly by titration of unlabeled proteins. Saad J, editor. PLoS ONE.

[111] Tedbury PR, Freed EO (2014). The role of matrix in HIV-1 envelope glycoprotein incorporation. Trends Microbiol.

[112] Chojnacki J, Staudt T, Glass B, Bingen P, Engelhardt J, Anders M (2012). Maturation-dependent HIV-1 surface protein redistribution revealed by fluorescence nanoscopy. Science.

[113] Zhu P, Chertova E, Bess J, Lifson JD, Arthur LO, Liu J (2003). Electron tomography analysis of envelope glycoprotein trimers on HIV and simian immunodeficiency virus virions. Proc Natl Acad Sci.

[114] Roy NH, Chan J, Lambele M, Thali M (2013). Clustering and mobility of HIV-1 env at viral assembly sites predict its propensity to induce cell-cell fusion. J Virol.

[115] Brugger B, Glass B, Haberkant P, Leibrecht I, Wieland FT, Krausslich H-G (2006). The HIV lipidome: a raft with an unusual composition. Proc Natl Acad Sci.

[116] Lorizate M, Sachsenheimer T, Glass B, Habermann A, Gerl MJ, Kräusslich H-G (2013). Comparative lipidomics analysis of HIV-1 particles and their producer cell membrane in different cell lines: Lipidomics of HIV-1 particles and producer plasma membranes. Cell Microbiol.

[117] Chan R, Uchil PD, Jin J, Shui G, Ott DE, Mothes W (2008). Retroviruses human immunodeficiency virus and murine leukemia virus are enriched in phosphoinositides. J Virol.

[118] Sezgin E, Levental I, Mayor S, Eggeling C (2017). The mystery of membrane organization: composition, regulation and roles of lipid rafts. Nat Rev Mol Cell Biol.

[119] Jouvenet N, Zhadina M, Bieniasz PD, Simon SM (2011). Dynamics of ESCRT protein recruitment during retroviral assembly. Nat Cell Biol.

[120] Baumgärtel V, Ivanchenko S, Dupont A, Sergeev M, Wiseman PW, Kräusslich H-G (2011). Live-cell visualization of dynamics of HIV budding site interactions with an ESCRT component. Nat Cell Biol.

[121] Prescher J, Baumgärtel V, Ivanchenko S, Torrano AA, Bräuchle C, Müller B (2015). Super-resolution imaging of ESCRT-proteins at HIV-1 assembly sites. Aiken C, editor. PLoS Pathog.

[122] Bleck M, Itano MS, Johnson DS, Thomas VK, North AJ, Bieniasz PD (2014). Temporal and spatial organization of ESCRT protein recruitment during HIV-1 budding. Proc Natl Acad Sci.

[123] Van Engelenburg SB, Shtengel G, Sengupta P, Waki K, Jarnik M, Ablan SD (2014). Distribution of ESCRT machinery at HIV assembly sites reveals virus scaffolding of ESCRT subunits. Science.

[124] Neil SJD, Zang T, Bieniasz PD (2008). Tetherin inhibits retrovirus release and is antagonized by HIV-1 Vpu. Nature.

[125] Van Damme N, Goff D, Katsura C, Jorgenson RL, Mitchell R, Johnson MC (2008). The interferon-induced protein BST-2 restricts HIV-1 release and is downregulated from the cell surface by the viral vpu protein. Cell Host Microbe.

[126] Lelek M, Di Nunzio F, Henriques R, Charneau P, Arhel N, Zimmer C (2012). Superresolution imaging of HIV in infected cells with FlAsH-PALM. Proc Natl Acad Sci.

[127] Brandenberg OF, Magnus C, Rusert P, Regoes RR, Trkola A (2015). Different infectivity of HIV-1 strains is linked to number of envelope trimers required for entry. Emerman M, editor. PLoS Pathog.

[128] Brandenberg OF, Magnus C, Regoes RR, Trkola A (2015). The HIV-1 entry process: a stoichiometric view. Trends Microbiol.

[129] Sattentau QJ (2010). Cell-to-cell spread of retroviruses. Viruses.

[130] Battistini A, Sgarbanti M (2014). HIV-1 latency: an update of molecular mechanisms and therapeutic strategies. Viruses.

[131] Malim MH, Emerman M (2008). HIV-1 accessory proteins—ensuring viral survival in a hostile environment. Cell Host Microbe.

[132] Dirk BS, Pawlak EN, Johnson AL, Van Nynatten LR, Jacob RA, Heit B (2016). HIV-1 Nef sequesters MHC-I intracellularly by targeting early stages of endocytosis and recycling. Sci Rep.

[133] Russell RA, Chojnacki J, Jones DM, Johnson E, Do T, Eggeling C (2017). Astrocytes resist HIV-1 fusion but engulf infected macrophage material. Cell Rep.

[134] Sougrat R, Bartesaghi A, Lifson JD, Bennett AE, Bess JW, Zabransky DJ (2007). Electron tomography of the contact between T cells and SIV/HIV-1: implications for viral entry. PLoS Pathog.

[135] Mengistu M, Ray K, Lewis GK, DeVico AL (2015). Antigenic properties of the human immunodeficiency virus envelope glycoprotein Gp120 on virions bound to target cells. Aiken C, editor. PLoS Pathog.

[136] Pereira CF, Rossy J, Owen DM, Mak J, Gaus K (2012). HIV taken by STORM: super-resolution fluorescence microscopy of a viral infection. Virol J.

[137] Pham S, Tabarin T, Garvey M, Pade C, Rossy J, Monaghan P (2015). Cryo-electron microscopy and single molecule fluorescent microscopy detect CD4 receptor induced HIV size expansion prior to cell entry. Virology.

[138] Peng K, Muranyi W, Glass B, Laketa V, Yant SR, Tsai L (2014). Quantitative microscopy of functional HIV post-entry complexes reveals association of replication with the viral capsid. eLife.

[139] Hulme AE, Kelley Z, Foley D, Hope TJ (2015). Complementary assays reveal a low level of CA associated with viral complexes in the nuclei of HIV-1-infected cells. Ross SR, editor. J Virol.

